# A randomized controlled trial of Shengji ointment combined with bromelain in promoting healing of tendon-exposed diabetic foot wounds: integrated 16S rDNA sequencing and metabolomics analysis

**DOI:** 10.3389/fphar.2025.1666278

**Published:** 2025-11-07

**Authors:** Xu Sun, Jielin Song, Junchao Sun, Zhaohui Zhang

**Affiliations:** 1 Department of Traditional Chinese Medicine Surgery, The Second Affiliated Hospital of Tianjin University of Traditional Chinese Medicine, Tianjin, China; 2 Tianjin Institute of Traditional Chinese Medicine Surgery, Tianjin, China; 3 TCM Institute of Sore and Ulcer, Tianjin University of Traditional Chinese Medicine, Tianjin, China; 4 Graduate School, Tianjin University of Traditional Chinese Medicine, Tianjin, China

**Keywords:** tendon-exposed wounds in diabetic foot, 16s rDNA sequencing, metabolomics, Shengji ointment, bromelain, Huafu Zaisheng method, diabetic foot ulcer

## Abstract

**Background:**

Diabetic foot tendon exposures are prone to infection, necrosis, and prolonged treatment cycle, not only hinder wound healing, but may also lead to amputation and even life-threatening. Utilizing Shengji ointment (a Traditional Chinese Medicine, TCM) combined with bromelain for treating diabetic foot tendon exposure wounds has demonstrated preliminary efficacy. However, the underlying mechanisms, as well as the changes in wound microbiota and metabolites before and after treatment, still warrant further investigation.

**Methods:**

This study used a randomized controlled trial design, with 60 patients randomly assigned to either the experimental group or the control group in a 1:1 ratio. The treatment cycle was 4 weeks. The experimental group was treated with Shengji ointment combined with bromelain, while the control group was treated with hydrocolloid dressing. The clinical efficacy of the two groups was evaluated through a controlled study, while wound exudates were collected only from the experimental group before and after treatment for 16S rDNA sequencing and metabolomics analysis to evaluate changes in wound microbiota and metabolites.

**Results:**

Following the treatment, the granulation tissue coverage, wound healing rate, and Maryland Foot Function Score in the experimental group were markedly superior to those of the control group. Post-treatment analysis revealed significant changes in the wound microbiota composition of the trial group, with a reduction in potential pathogenic bacteria, including Erysipelatoclostridium, Lachnoclostridium, Pontimonas, Hydrogenovibrio, and Aquabacterium, alongside an increase in beneficial bacteria, such as Cetobacterium and Allisonella. Furthermore, 4,034 differential metabolites were identified, with 1907 upregulated and 2,127 downregulated, involving key metabolic pathways such as phenylpropanoid biosynthesis, tyrosine metabolism, and amino acid biosynthesis. Correlation analysis indicated a strong negative association between Pontimonas, Hydrogenovibrio, Aquabacterium, and the majority of the differential metabolites.

**Conclusion:**

The Shengji ointment combined with bromelain has shown notable effectiveness in treating diabetic foot wounds with tendon exposure by modulating microbial composition (reducing pathogenic bacteria and increasing beneficial bacteria) and optimizing the metabolic environment (targeting key metabolic pathways), providing valuable insights for further exploration of its mechanisms.

## 1 Introduction

Diabetic foot (DF), one of the most prevalent and serious complications of diabetes, is frequently associated with lower limb neuropathy and peripheral vascular disease (Ogbera et al., 2008; Baroni et al., 2014; Bowling et al., 2015; Mariam et al., 2017; Huang et al., 2024). It typically manifests as skin infections, ulcers, and deep tissue damage below the ankle, potentially leading to foot gangrene or even amputation in severe cases (Lipsky, 2024; Dalla Paola and Faglia, 2006; Stancu et al., 2022; Armstrong et al., 2023; Matijević et al., 2023). Diabetic patients face a lifetime risk of 15%–25% for developing diabetic foot ulcers (DFU). For those with a history of previous foot injuries or infections, the incidence of DFU can ascend to 19%–34%, with approximately 17% of such patients requiring amputation (Armstrong et al., 2017; Lipsky et al., 2020). Diabetic foot tendon exposure typically occurs in the mid to late stage of the ulcerative period, with a Wagner classification ranging from 2 to 5 and a TEXAS classification of grade 3 (Wagner, 1981; Lavery et al., 1996). It is manifested as the gradual degeneration and necrosis of the tendon, along with the spread of infection along the tendon towards the proximal end, ultimately resulting in amputation or death, imposing substantial burdens on individuals, healthcare systems, and economies worldwide (Boulton et al., 2005; Raghav et al., 2018; Teobaldi et al., 2018; Behzadifar et al., 2023; McDermott et al., 2023; Zhao et al., 2023a). The current prevailing treatment modality involves surgical excision of the exposed necrotic or degenerated tendon. However, this approach may lead to a partial loss of foot function and inflict damage on the newly formed collateral microcirculation in the surrounding tissues (Sun et al., 2024). Consequently, there is an imperative need to explore a mild and efficacious tendon removal approach for optimizing treatment strategies.

In Traditional Chinese Medicine clinical practice, the Huafu Zaisheng method involves the application of bromelain combined with Shengji ointment to treat diabetic foot wounds with exposed tendons (Zhang et al., 2012a; 2012b). This approach selectively removes degenerated and necrotic tendon tissue without harming healthy structures, thereby promoting the formation of granulation tissue to cover the exposed tendons. Ultimately, it accelerates wound healing while contributing to the preservation of foot functionality. Our previous study has demonstrated that the method of Huafu Zaisheng facilitates tissue regeneration and *in situ* repair (Zhang et al., 2012c). The exudate from the wound is the outcome of material alterations resulting from the action of the active ingredients of the drug on the wound, representing variations in the microenvironment of the wound (Löffler et al., 2013; Widgerow et al., 2015; Junka et al., 2017; Dong et al., 2022; Gao et al., 2022; Gould and Mahmoudi, 2024). Consequently, investigating the patterns and trends of exudate changes holds significant importance for uncovering the treatment mechanisms. The study suggests that this approach may promote wound healing by modifying wound pH levels and increasing the concentrations of amino acids, proteins, and lysozyme in the wound exudate (Zhang et al., 2011). However, the specific alterations in the microbial community and the underlying metabolic mechanisms in wound exudate before and after intervention with the Huafu Zaisheng method remain inadequately understood.

This study employed 16S rDNA sequencing in conjunction with non-targeted metabolomics to examine the alterations in wound microbiota and metabolites in patients before and after treatment. The objective was to ascertain the correlation between these changes and to elucidate the potential mechanism through which this method facilitates wound healing in diabetic foot tendon exposure. This study was registered at the Chinese Clinical Trial Registry (ChiCTR2000039327) on 23 October 2020.

## 2 Materials and methods

### 2.1 Research design and source

This study was a randomized controlled trial conducted from 1 December 2020, to 31 December 2021, designed to evaluate the clinical efficacy and potential mechanism of the Huafu Zaisheng method in treating patients with DFU. All participants were recruited from the Second Affiliated Hospital of Tianjin University of Traditional Chinese Medicine. Prior to enrollment, each patient voluntarily provided written informed consent. The research protocol received approval from the hospital’s Ethics Committee (Approval number: 2020-006-01).

### 2.2 Inclusion and exclusion criteria

#### 2.2.1 Inclusion criteria

Patients who met the following criteria were included. (1) Patients who met the diagnostic criteria of diabetic foot and Wagner grade 3–4 with tendons exposed; (2) Age between 18 and 85 years; (3) Fasting blood glucose ≤10 mmol/L; (4) Targeted ulcer debridement area between 1 and 20 cm^2^ (for patients with multiple lesions, the largest ulcer will be the target lesion); (5) An ankle‒brachial index ≥0.5 on the side of the limb with the ulcer; (6) The ulcer has blood, pus, or sticky secretions; (7) Voluntary participation and signing an informed consent form.

#### 2.2.2 Exclusion criteria

Patients who met any of the following criteria were excluded. (1) DFU caused by electrical, chemical, radioactive, neoplastic, or varicose veins, among other reasons, or malignant lesions within the ulcer; (2) There were clinical signs of a systemic infection, such as cellulitis, fever, increased white blood cells, or a positive bacterial culture; (3) Severe uncontrolled hypertension with a systolic blood pressure of ≥160 mmHg or a diastolic blood pressure of ≥110 mmHg; (4) Serum albumin <28 g/L; (5) Hemoglobin <90 g/L; (6) Platelet count <50 × 10^9^/L; (7) Severe heart, liver, or kidney injury, in which case medical treatment may seriously affect patient safety; (8) Women who are pregnant, lactating, recently pregnant, or planning to get pregnant in the near future; (9) Patients with cognitive impairment who could not fully understand the research content or give informed consent; (10) Patients who were allergic to some components of the study drug; (11) Participation in other types of clinical trials within the last month; (12) Poor compliance, inability to complete the study or failure to comply with the study regulations.

### 2.3 Grouping and intervention

#### 2.3.1 Grouping and sample size calculation

Based on preliminary clinical trial results demonstrating efficacy rates of 83% (treatment group) and 50% (control group), we performed sample size estimation using PASS software (version 21.0). With parameters set at a two-sided α = 0.05, 80% statistical power, and 1:1 allocation ratio, the initial calculation yielded a requirement of 58 participants. Accounting for a potential 10% dropout rate, the final estimated sample size was determined as 64 cases (32 per group).

This study employed a parallel-group randomized controlled trial design. Using the minimization method, a 1:1 dynamic randomization was implemented via the MagMinDA system, with the allocation bias probability set at 1.0 to rigorously balance baseline characteristics between groups. The random allocation sequence was dynamically generated in real-time by the system, with automated encrypted group assignment to guarantee allocation concealment. Neither participants nor researchers were blinded to treatment assignments after randomization. Eligible participants were enrolled by independent study coordinators who had no access to the allocation sequence. Wound exudate samples were collected from the treatment group before (PRE_T) and after (POST_T) therapy for subsequent 16S rDNA sequencing and untargeted metabolomics analysis.

#### 2.3.2 Intervention measures

In the experimental group (Huafu Zaisheng method), bromelain powder was evenly applied to the exposed tendon surface of the wound, followed by external application of Shengji ointment-coated defatted cosmetic cotton dressing. The control group received standard wound care using Comfeel^®^ Hydrocolloid Dressing. The treatment duration for both groups was 4 weeks, with daily dressing changes. Bromelain was obtained from Olive Branch Pharmaceutical Co., Ltd. (Shantou, Guangdong Province, China; specification: 10,000 units; National Drug Approval No.: H44024825; Batch No.: 200,913). Shengji ointment was purchased from Tianjin Darentang Jingwanhong Pharmaceutical Co., Ltd. (Tianjin, China; specification: 30 g/box; National Drug Approval No.: Z12020345; Batch No.: 206,600). Shengji ointment contains Gypsum Fibrosum, Crinis Carbonisatus, Rehmannia glutinosa, Angelica sinensis, calamine, and beeswax as its key components. Comfeel^®^ Hydrocolloid Dressing (debridement gel, 20 g/tube; Coloplast A/S, Denmark; China Medical Device Import Registration Certificate No.: SFDA(I)2008-3640,515; Batch No.: 103,579).

### 2.4 Outcome indicators

#### 2.4.1 Primary outcome indicator

The primary outcome indicator, granulation tissue coverage rate, is calculated as the percentage of the area covered by granulation tissue relative to the total wound area (Wang et al., 2023; 2024).

#### 2.4.2 Secondary outcome indicator

The secondary outcome indicators include the wound healing rate and the Maryland Foot Function Score. The wound healing rate was defined as the percentage of the difference between the original wound area and the unhealed area relative to the original wound area (Li et al., 2024; Shahat et al., 2024; Thng et al., 2024; Tomoi et al., 2024; Xu et al., 2024). The Maryland Foot Function Score was used to evaluate foot function, including the presence or absence of pain. The maximum score is 100 points, with scores >89 points indicating excellent function, 75–89 points indicating good function, 50–74 points indicating fair function, and <50 points indicating poor function.

### 2.5 Collection of exudate from wound

A non-invasive technique was utilized. A sterile cotton swab was rotated for 5 s at the granulation tissue’s center to ensure sufficient exudate collection. The swab was subsequently placed in sterile packaging and stored at −80 °C until further analysis.

### 2.6 16S rDNA analysis

Total microbial DNA was isolated from wound exudate samples using the CTAB method. DNA quality was assessed by 0.8% agarose gel electrophoresis, and concentration was measured using NanoDrop spectrophotometry. The V3-V4 hypervariable regions of bacterial 16S rDNA genes were amplified using primers 341F/806R under standard PCR conditions. Amplification products were verified by 2% agarose gel electrophoresis alongside negative controls. After quantification and purification, equimolar pools were prepared for Illumina NovaSeq 6,000 sequencing (2 × 250 bp paired-end). Raw sequences were processed using the DADA2 pipeline for quality filtering (Q-score ≥20), chimera removal, and ASV generation. Taxonomic classification was performed against the SILVA database (v138) using a Naïve Bayes classifier with 99% confidence threshold. Microbial diversity was assessed using Shannon and Chao1 indices (alpha diversity) and Bray-Curtis dissimilarity (beta diversity). Differential abundance analysis was conducted using Mann-Whitney U tests with FDR correction (p < 0.05). Biomarker identification employed LEfSe analysis. Functional potential was predicted using PICRUSt2 based on MetaCyc pathways.

### 2.7 Untargeted metabolomics analysis

Metabolites were extracted from wound exudate samples using ice-cold methanol (4:1 v/v methanol:sample) with overnight protein precipitation at −20 °C. After centrifugation (12,000 × g, 10 min, 4 °C), supernatants were concentrated under nitrogen gas and stored at −80 °C until analysis. Chromatographic separation was performed on a UHPLC system (ACQUITY BEH C18 column; 100 × 2.1 mm, 1.8 µm) with a 20-min gradient (5%–95% acetonitrile/0.1% formic acid) at 0.4 mL/min. Metabolites were analyzed via Q Exactive HF-X Orbitrap MS in both ionization modes (70,000 resolution, m/z 70–1,050), with data-dependent MS/MS fragmentation (top 3 precursors, HCD at 28 eV). System stability was monitored using pooled QC samples injected every 10 runs. Raw LC-MS/MS data were processed in XCMS (signal-to-noise >5, RT deviation <0.3 min), with CAMERA for adduct annotation. Metabolites were identified by mass matching (±5 ppm) against HMDB/KEGG (Level 1) and MS/MS spectral matching against an in-house library of 800 standards (Level 2; mass error <0.02 Da, dot product >70%). PCA validated QC stability, while OPLS-DA (VIP≥1.0, P < 0.05) identified differential metabolites (q < 0.05, fold change >2). Enriched KEGG pathways (P < 0.01) were inferred via clusterProfiler.

### 2.8 Statistical analysis

Results are expressed as mean ± standard deviation (SD) or median with interquartile range (IQR). Normality (Shapiro-Wilk test) and variance homogeneity (Levene’s test) were assessed. Normally distributed data were compared using independent t-tests, while non-normal data were analyzed with Mann-Whitney U tests. Spearman’s correlation was used to examine relationships between metabolites and microbial genera. For handling missing data due to patient dropouts, complete case analysis was performed as primary approach, where participants with missing follow-up data were excluded from analysis. All analyses were conducted in GraphPad Prism 7.0, with p < 0.05 considered statistically significant.

## 3 Results

**FIGURE 1:**
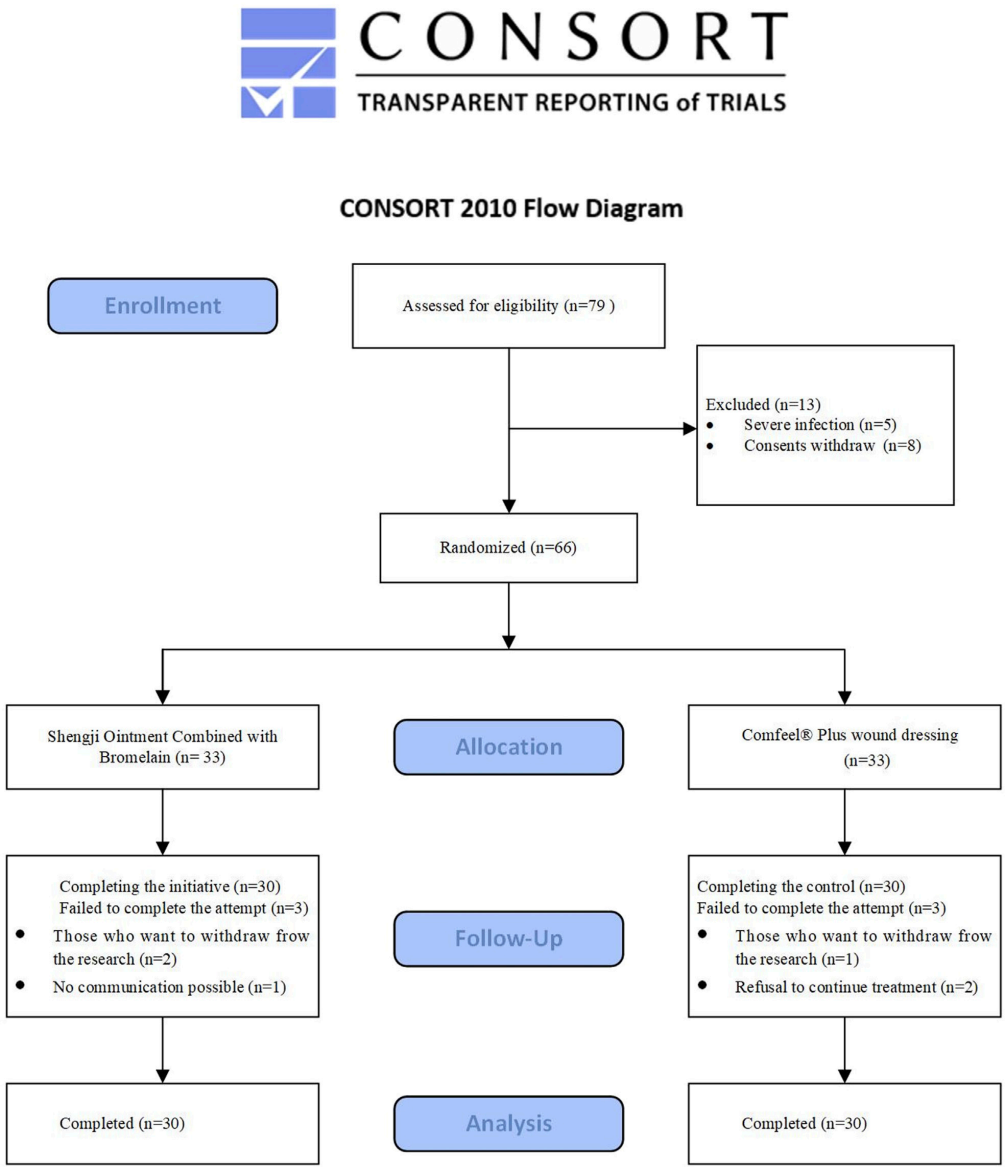
CONSORT flowchart of participant enrollment.

During the study, 79 individuals were assessed for eligibility, of whom 13 were excluded. Thirty-three participants were enrolled in each group, and 3 from each group discontinued the intervention. Ultimately, 30 patients per group completed the treatment (Figure 1).

### 3.1 Clinical characteristics and efficacy analysis

**FIGURE 2:**
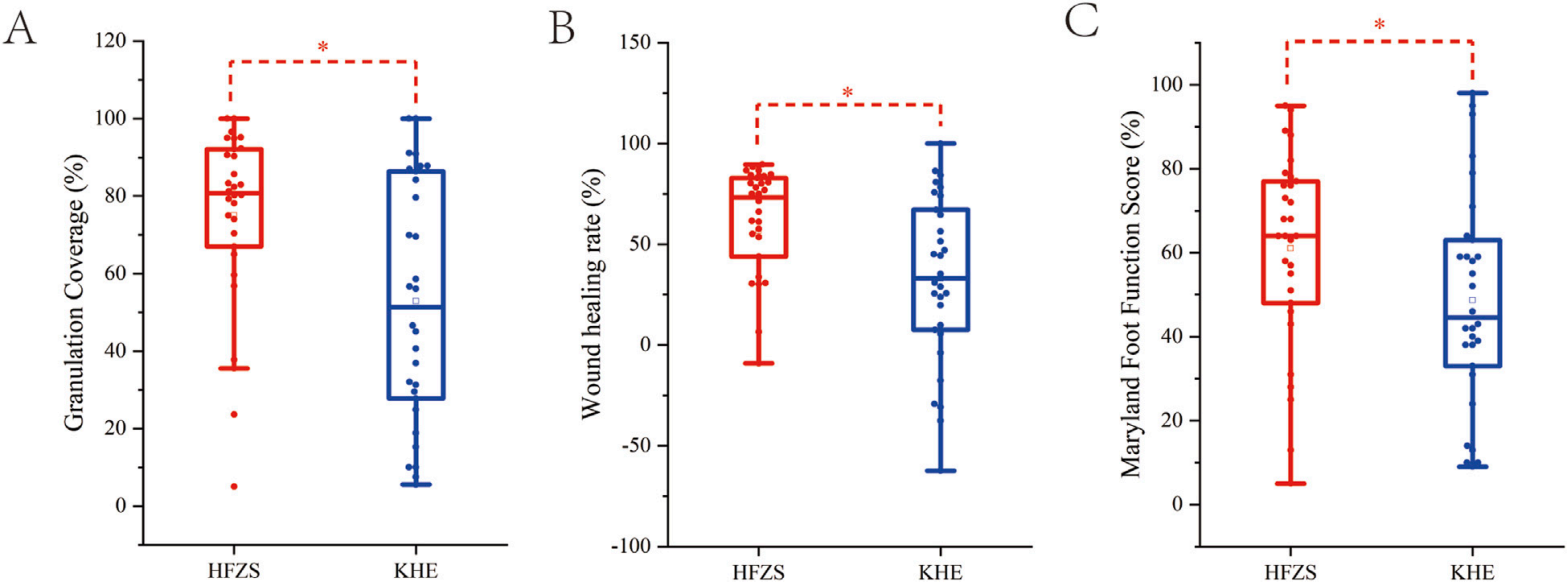
Comparison of therapeutic efficacy between two groups. **(A)** The granulation tissue coverage rates after treatment. **(B)** The wound healing rates after treatment. **(C)** Maryland foot function score after treatment. HFZS: Huafu Zaisheng method. KHE: Comfeel^®^ Plus wound dressing. “*” indicates *P* < 0.05.

An analysis of baseline characteristics, including gender, age, ankle-brachial index, Wagner grading, and disease location, revealed no statistically significant differences between the two groups (*P* > 0.05) (Table 1), confirming the comparability of treatment outcomes. After 4 weeks of treatment, the experimental group demonstrated significantly higher granulation tissue coverage rate, wound healing rate, and Maryland Foot Function Score compared to the control group (*P < 0.05*). As the data were non-normally distributed, results are presented as median (IQR) (see Table 2; Figure 2).

**TABLE 1:** Baseline characteristics analysis of the two groups.

Variables	Observation group N = 30	Control group N = 30	X^2^/t/Z	*P* value
Sex				
Male [n(%)]	18 (60.00)	12 (40.00)	2.40	0.12
Female [n(%)]	12 (40.00)	18 (60.00)		
Age, mean (SD), years	62.73 (10.20)	66.23 (11.15)	1.27	0.21
ABI, Median (IQR)	0.8 (0.13)	0.8 (0.03)	473.5	0.71
Wagner grading				
Grade 3 [n(%)]	17 (56.67)	17 (56.67)	0	1
Grade 4 [n(%)]	13 (43.33)	13 (43.33)		
Location of disease				
Dorsal [n (%)]	9 (30.00)	3 (10.00)	4.35	0.11
Plantar [n (%)]	7 (23.33)	12 (40.00)		
Toe [n (%)]	14 (46.67)	15 (50.00)		

ABI: Ankle-Brachial Index.

**TABLE 2:** Clinical efficacy analysis of the two groups.

Outcomes	Visit	Observation group N = 30	Control group N = 30	Z	*P* value
Wound granulation, Median (IQR), %	Baseline	15.00 (34.75)	39.5 (52.50)	−1.47	0.14
	4 weeks	80.77 (25.66)	51.33 (59.45)	−2.60	0.01
	Difference efficacy	52.88 (43.58)	18.39 (33.91)	−3.62	0.01
Wound healing rate, Median (IQR), %	4 weeks	73.21 (41.77)	33.11 (61.86)	−2.73	0.01
Maryland foot function score, Median (IQR), point	Baseline	54.00 (28.50)	42.00 (36.75)	−0.851	0.395
	4 weeks	64.00 (29.75)	44.50 (30.75)	−2.085	0.037
	Difference efficacy	11.50 (13.75)	0.50 (4.25)	−3.63	0.001

### 3.2 16S rDNA analysis of wound microbiota

#### 3.2.1 Analysis of diversity and composition of wound microbiota before and after treatment with Huafu Zaisheng method

**FIGURE 3:**
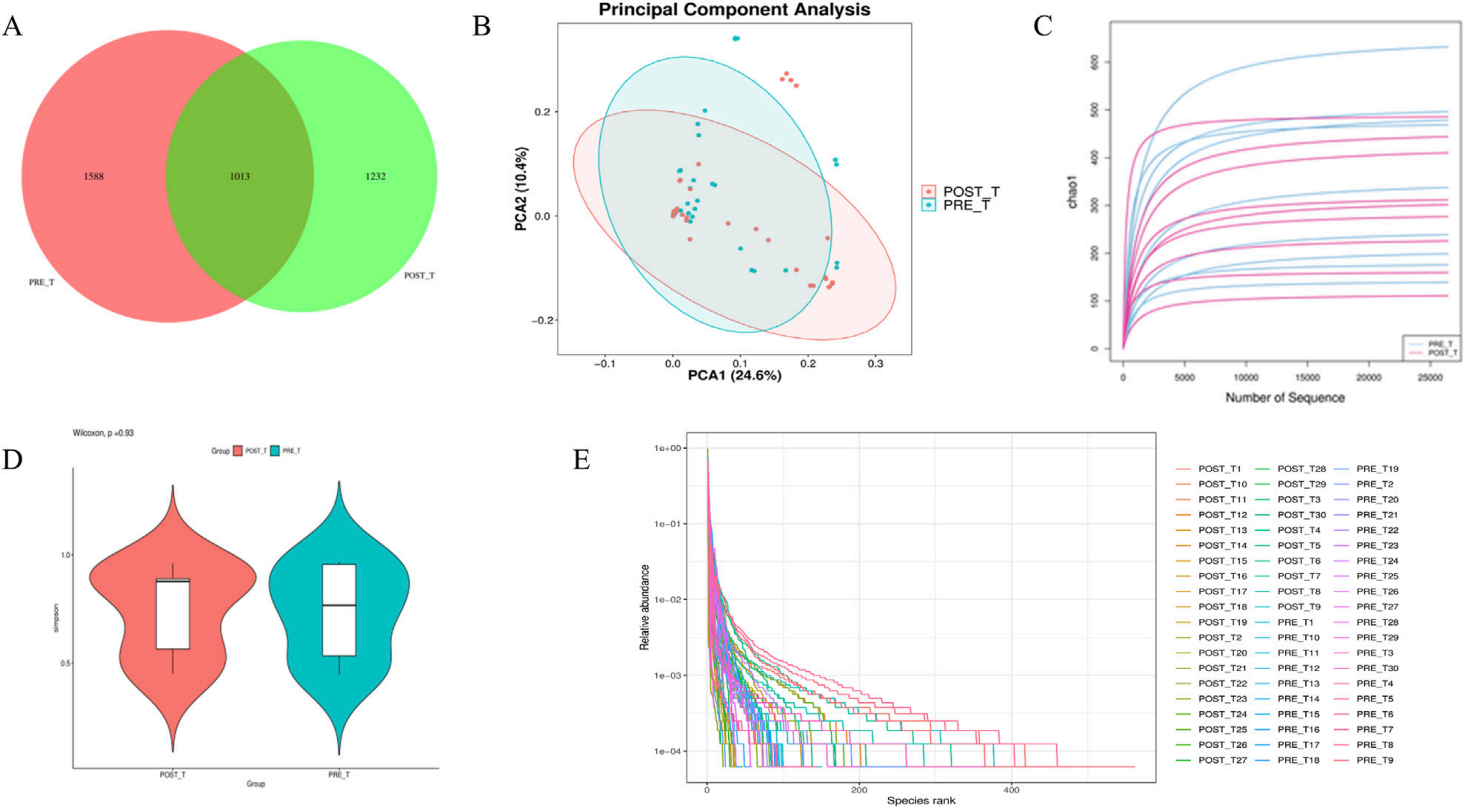
Diversity and composition analysis of wound microbiota before and after Treatment. **(A)** ASV Distribution Venn Diagram. **(B)** PCA plot illustrating beta diversity differences. **(C)** Rarefaction curves of Simpson index showing sequencing depth adequacy. **(D)** Representative Chao1 index for alpha diversity. **(E)** Rank Abundance curve depicting species richness and dominance.

A Venn diagram was generated based on the ASV abundance table (Figure 3A). The analysis identified 1,013 shared ASVs between the two groups, with 1,588 ASVs unique to the pre-treatment group and 1,232 ASVs unique to the post-treatment group. These results indicate that the composition of the wound microbiota was altered following treatment with the Huafu Zaisheng method. To evaluate the α-diversity of wound microbiota before and after treatment, indices including Chao1, Observed species, Goods coverage, Shannon, Simpson, and Pielou e were analyzed. The results showed no significant differences in these indices between the pre- and post-treatment groups (p > 0.05). For simplicity, the Chao1 index, as a representative metric, is presented in Figure 3D, while the results for the other indices are provided in the Supplementary Material. The Simpson index rarefaction curve indicates that as the sampling depth increases, the curve approaches a plateau, suggesting that the sequencing depth was sufficient to capture the majority of microbial diversity (Figure 3C). The Rank Abundance curve spans a wide range on the horizontal axis, indicating high species richness. Meanwhile, the curve exhibits a certain degree of steepness on the vertical axis, suggesting the presence of dominant microbial species in the community (Figure 3E). The beta diversity of wound microbiota before and after treatment was analyzed using PCA. Differences in the shape and distribution observed in the PCA suggest potential variations in microbial community composition between the pre- and post-treatment groups (Figure 3B).

#### 3.2.2 Hierarchical changes in bacterial abundance after Huafu Zaisheng method treatment

**FIGURE 4:**
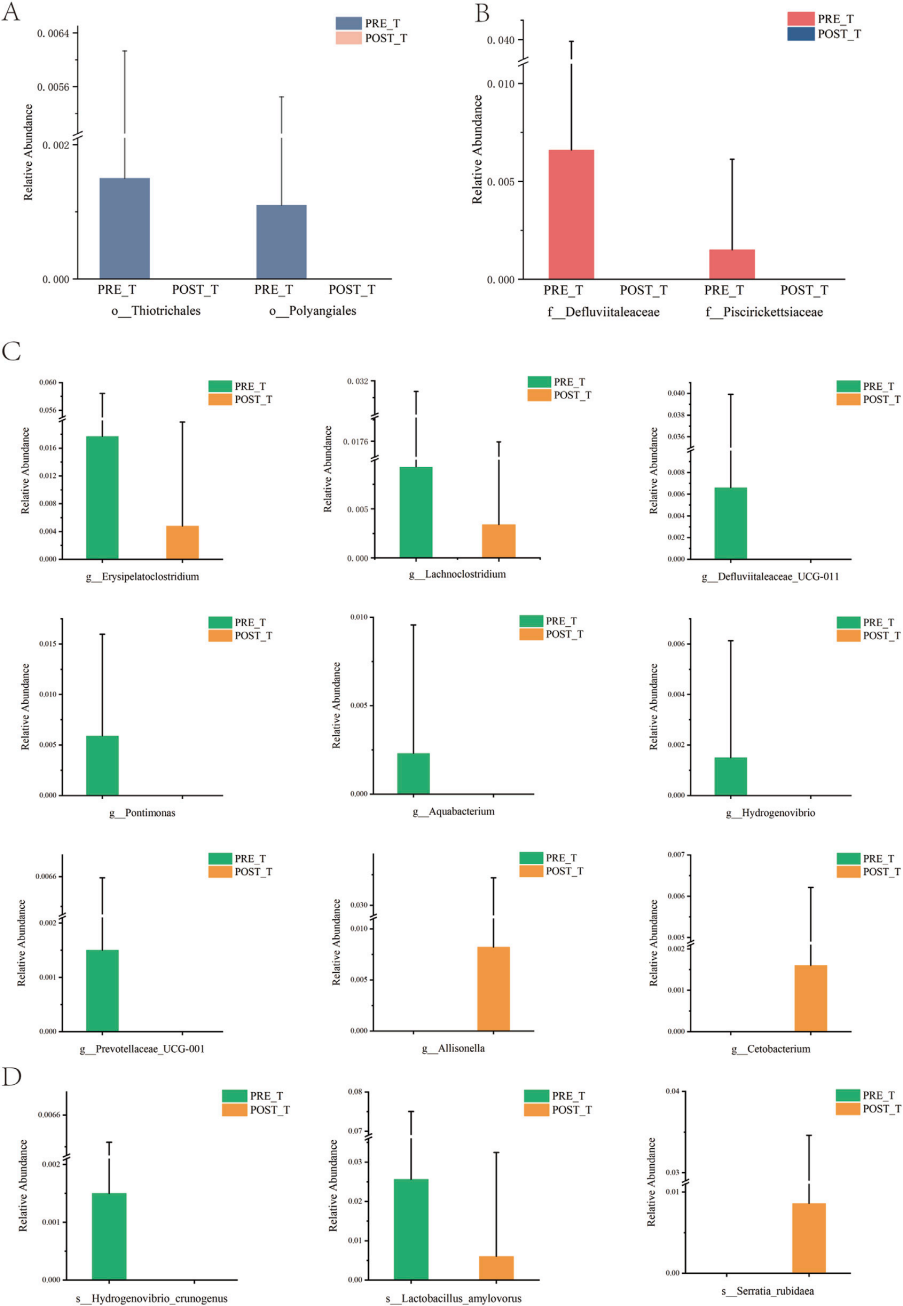
An analysis of the disparities in the wound microbiome before and after treatment at various classification levels. **(A)** Relative abundance of species at the order level. **(B)** Relative abundance of species at the family level. **(C)** Relative abundance of species at the genus level. **(D)** Relative abundance of species at the species level.

Differential analysis was performed for species at each taxonomic level, and significant differential species (P < 0.05) were identified. At the mesophyll level, Thiotrichales and Polyangiales showed a significant decrease after treatment compared to before treatment (Figure 4A). At the family level, Defluviitaleaceae and Piscirickettsiaceae exhibited a significant decrease after treatment compared to before treatment (Figure 4B). At the genus level, after excluding unclassified genera, a significant decrease was observed in Erysipelatoclostridium, Lachnoclostridium, Defluviitaleaceae_UCG-011, Pontimonas, Aquabacterium, Hydrogenovibrio, and Prevotellaceae_UCG-001 after treatment compared to before treatment, while Cetobacterium and Allisonella significantly increased after treatment (Figure 4C). At the species level, after removing unclassified taxa, a significant decrease was observed in Hydrogenovibrio crunogenus and *Lactobacillus* amylovorus after treatment, while *Serratia* rubidaea showed an increase (Figure 4D).

**FIGURE 5:**
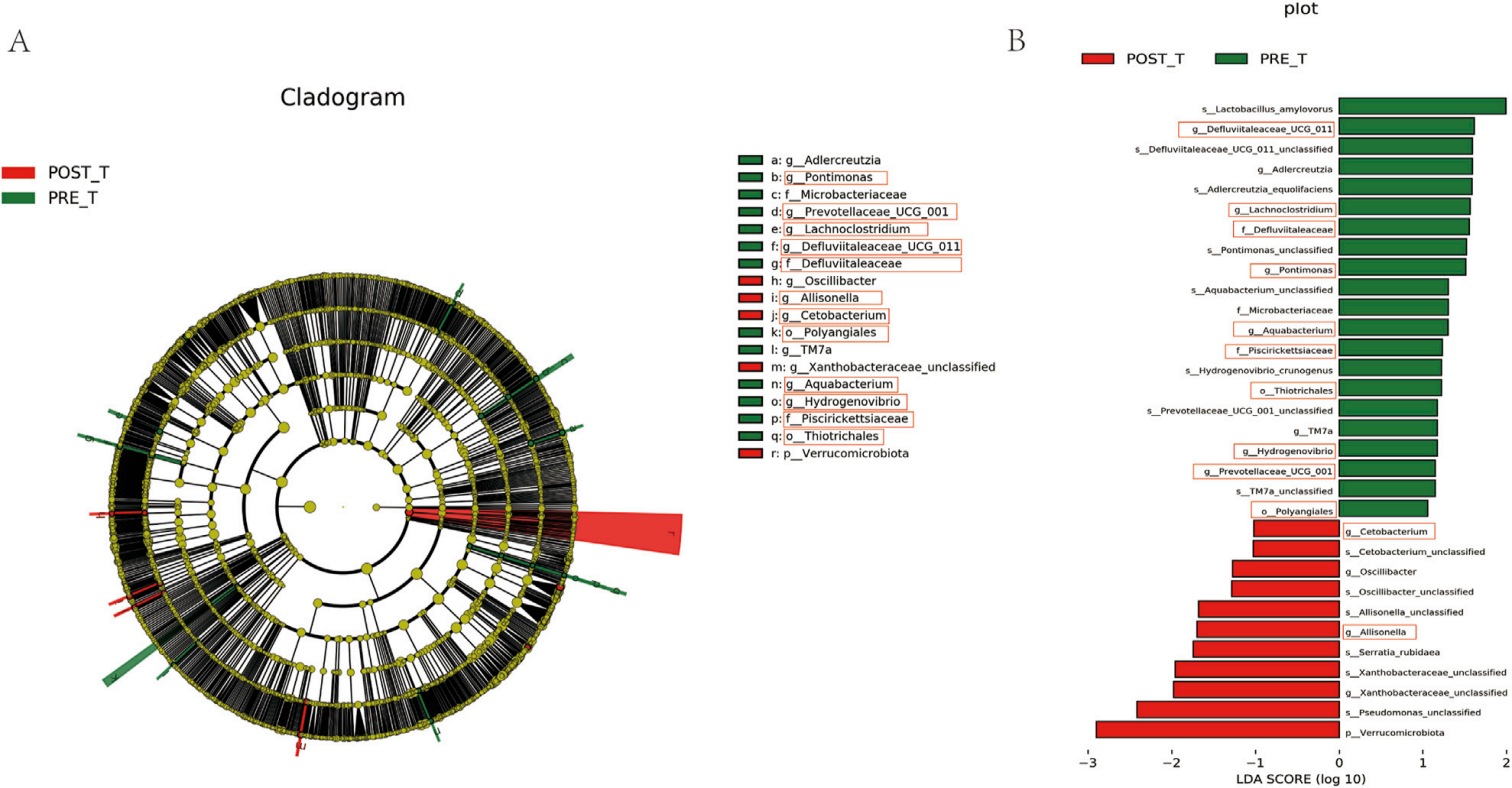
Differential species analysis and LDA effect size visualization. **(A)** Differential species annotation cladogram. **(B)** Histogram of LDA Effect Size.

LEfSe was applied to identify differential bacterial taxa with a Linear Discriminant Analysis (LDA) score >1 and p < 0.05 between pre-treatment and post-treatment groups. The cladogram (Figure 5A) illustrates the phylogenetic relationships between distinct microbiota at the phylum to genus level. The letters “p”, “c”, “o”, “f”, and “g” represent phyla, class, order, family, and genus, respectively. The LEfSe analysis results (Figure 5B) indicate that the predominant bacteria in the pre-treatment group were Thiotrichales, Polyangiale, Defluviitaleaceae, Piscirickettsiaceae, Lachnoclostridium, Defluviitaleaceae_UCG-011, Pontimonas, Aquabacterium, Hydrogenovibrio, and Prevotellaceae_UCG-001. These bacteria may be associated with pre-treatment dysbiosis. Following treatment, the group exhibited a notable increase in Cetobacterium and Allisonella, which indicates that the treatment may facilitate wound healing by reducing the prevalence of potential pathogens and promoting the enrichment of bacteria associated with tissue repair.

#### 3.2.3 Functional prediction of wound microbiome

**FIGURE 6:**
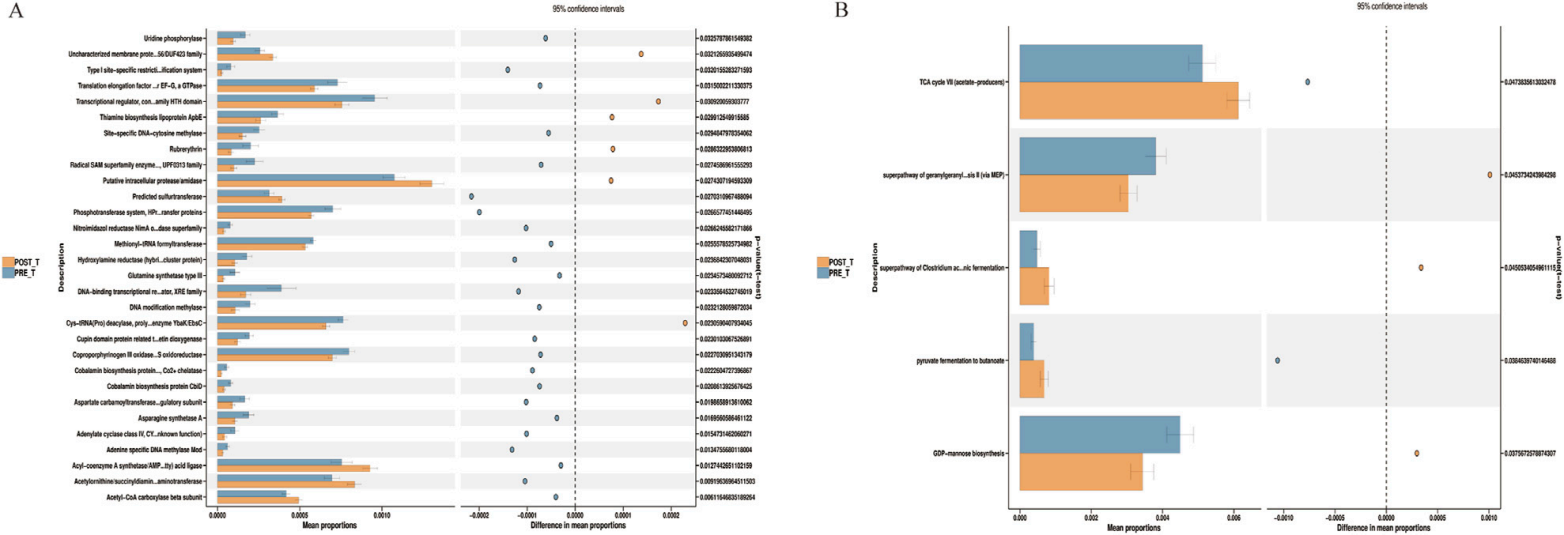
Differential microbial function analysis based on COG **(A)** and pathway **(B)**.

Based on PICRUSt2 functional predictions, we compared the metabolic functions of the wound microbiome before and after treatment with Huafu Zaisheng method. Analysis based on the COG database indicated that post-treatment, there was a significant downregulation in functions including uridine phosphorylase, asparagine synthetase A, and cobalamin biosynthesis protein (*P* < 0.05) (Figure 6A). Furthermore, KEGG pathway analysis demonstrated that post-treatment, there was a significant upregulation in functions such as the tricarboxylic acid cycle VII and pyruvate fermentation to butanoate, while the function of mannose biosynthesis was significantly downregulated (*P* < 0.05) (Figure 6B).

### 3.3 Untargeted metabolomics analysis

#### 3.3.1 Data quality control

**FIGURE 7:**
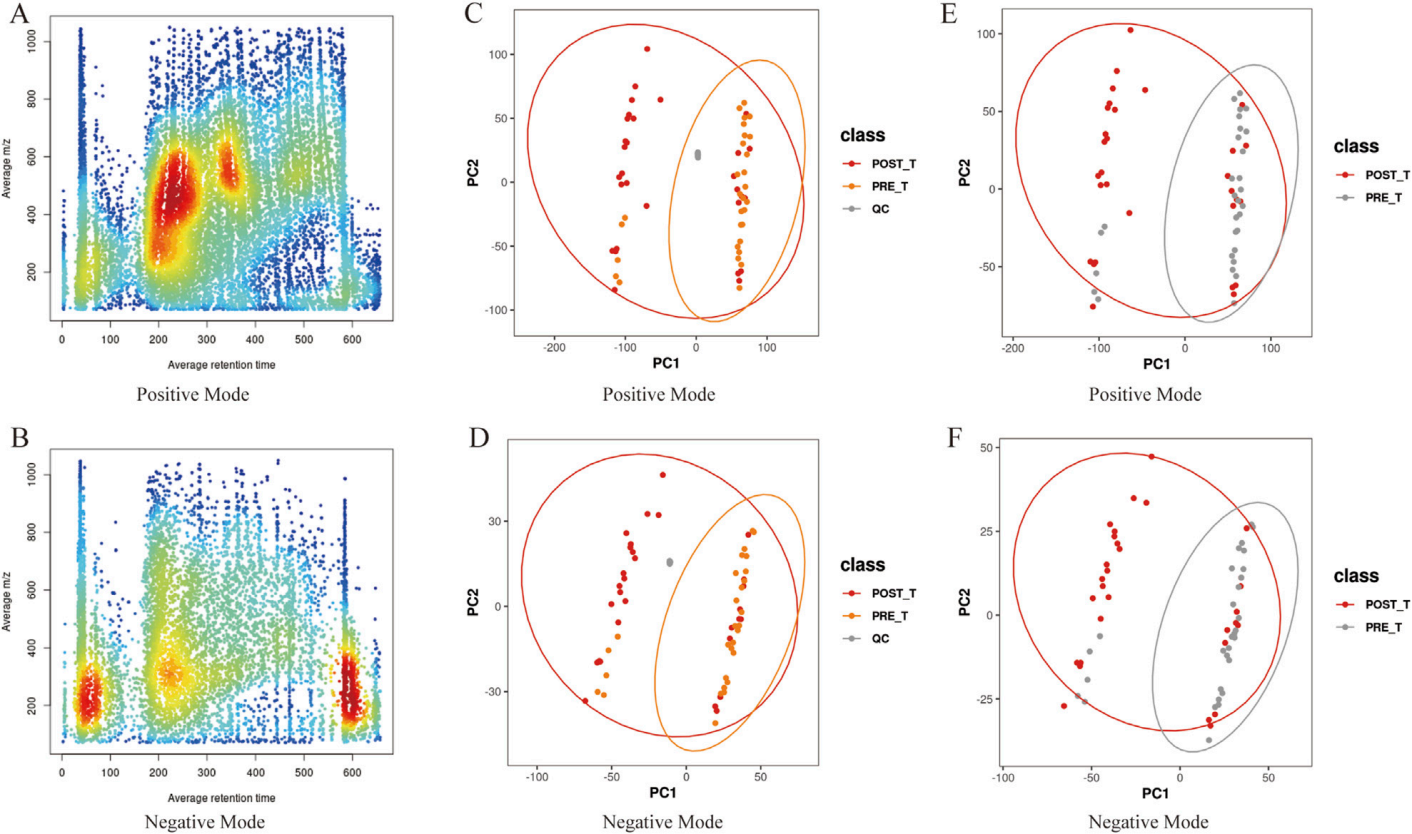
Data quality analysis. **(A,B)** The m/z-rt distribution plot of positive/negative ion metabolites, with rt on the x-axis and m/z on the y-axis. **(C,D)** PCA plots of positive/negative ions including quality control (QC) samples. **(E,F)** PCA plots of positive/negative ions for pre- and post-treatment groups.

The metabolite data were acquired using a Q-Exactive high-resolution mass spectrometer with a resolution greater than 30,000. For multi-sample analysis, peak alignment was performed based on small variations in mass-to-charge ratio (m/z) (±0.01) and retention time (rt) (±0.5 min) to ensure accurate quantification of metabolites. Data processing was carried out using XCMS software, and metabolite abundance was visualized by color intensity, with darker colors indicating higher metabolite concentrations. In positive ion mode, the m/z range of 200–600 and retention time between 200 and 400 s exhibited the highest number of detected peaks, suggesting a higher abundance of metabolites in this range and confirming the stability and reliability of the data quality (Figure 7A). In negative ion mode, the m/z range of 0–400, with peak intensities observed in several retention time windows (e.g., 0–100s, 200–300s, and 550–650s), reflected the variation in metabolite abundances across different metabolites (Figure 7B). Overall, the peak alignment ensures precise metabolite quantification, while the distinct peak patterns across different time windows and m/z ranges confirm the stability, consistency, and high reproducibility of the data. These results provide a solid foundation for further metabolomics analysis.

PCA was performed to analyze the metabolite ions identified before and after Huafu Zaisheng method. The quality control samples clustered tightly, indicating stable detection quality (Figures 7C,D). In the PCA plot, some overlap of samples was observed, which may be attributed to biological variability and technical noise. Nevertheless, the metabolite trend plots show a clear separation between the pre- and post-treatment samples, suggesting the presence of metabolic differences between the two groups (Figures 7E,F). These findings provide a reliable foundation for further analysis, indicating that the treatment induced changes in the metabolite profile.

#### 3.3.2 Analysis of differential metabolites

**FIGURE 8:**
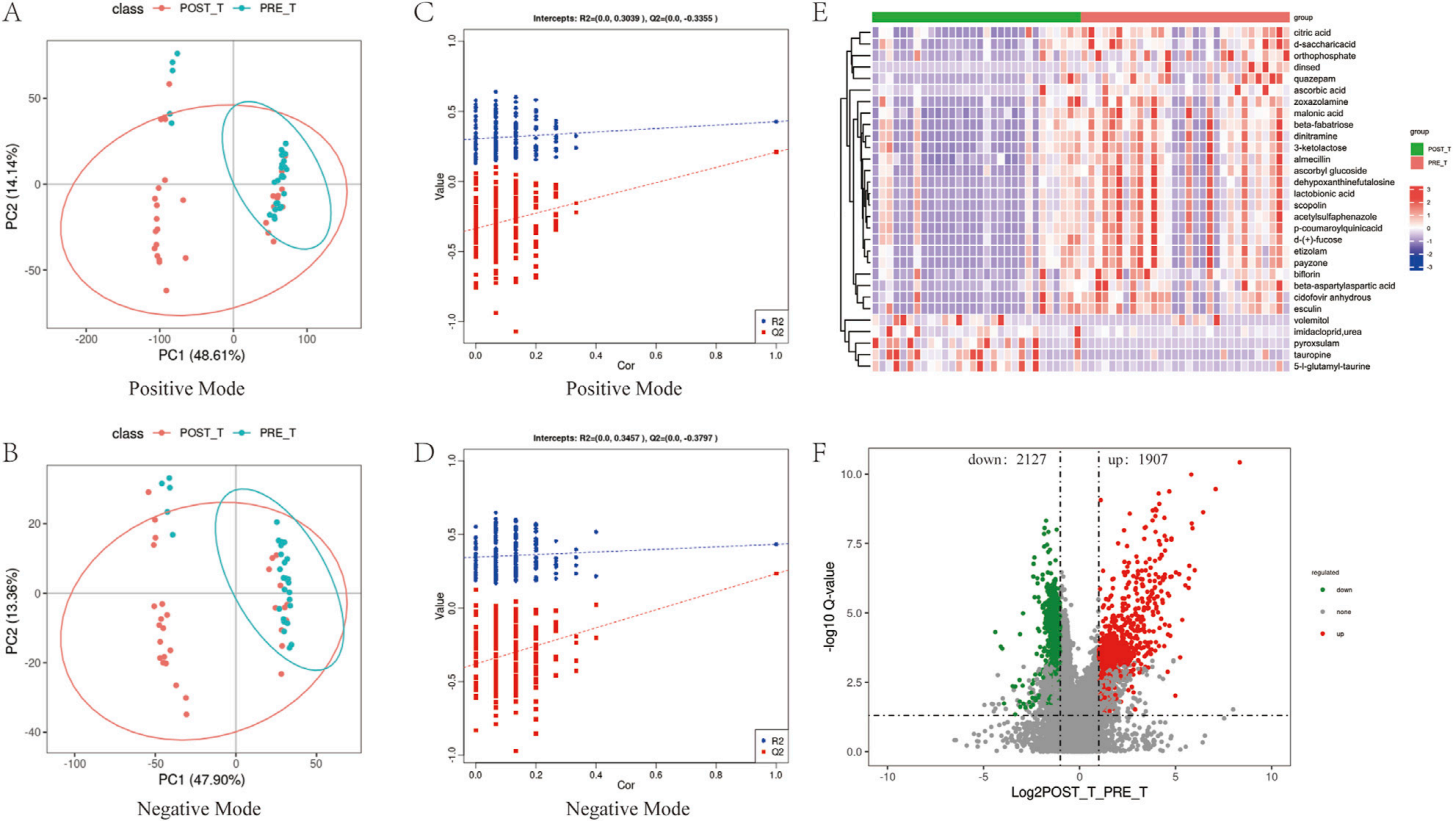
Analysis of differential metabolites. **(A,B)** PLS-DA score plots of positive and negative ions before and after treatment. **(C,D)** Permutation test results of the PLS-DA score plots for positive and negative ions. **(E,F)** Heatmap of the top 30 most significant differential metabolites and volcano plot representing the complete set of differential metabolites.

A PLS-DA was performed to model and predict the metabolic expression profiles of samples before and after treatment with the Huafu Zaisheng method. The Variable Importance in Projection (VIP) scores were used to assess the contribution of individual metabolites to the classification. Metabolites with VIP scores ≥1.0 were considered to significantly contribute to the classification and were identified as potential metabolic biomarkers. Separate PLS-DA models were established for each group, and the robustness of the models was validated by 200 permutation tests, evaluating the statistical significance of the explanatory power (R^2^) and predictive ability (Q^2^) of the models. The PLS-DA score plots (Figures 8A,B) showed partial overlap in the distribution of samples before and after treatment but revealed a clear separation trend overall, indicating significant metabolic differences between the two groups. The permutation test results (Figures 8C,D) demonstrated that the R^2^ values were consistently higher than the Q^2^ values, and the regression line of Q^2^ intersected the y-axis below zero. These findings indicate that the model exhibits good predictive performance without overfitting. The robustness of the model further supports the reliability and significance of the identified differential metabolites in distinguishing between the two groups. Compared to the pre-treatment group, a total of 4,034 differential metabolites were identified after treatment, including 1,907 upregulated and 2,127 downregulated metabolites (Figure 8F). A heatmap of the top 30 differential metabolites is presented in Figure 8E.

#### 3.3.3 Analysis of differential pathways

**FIGURE 9:**
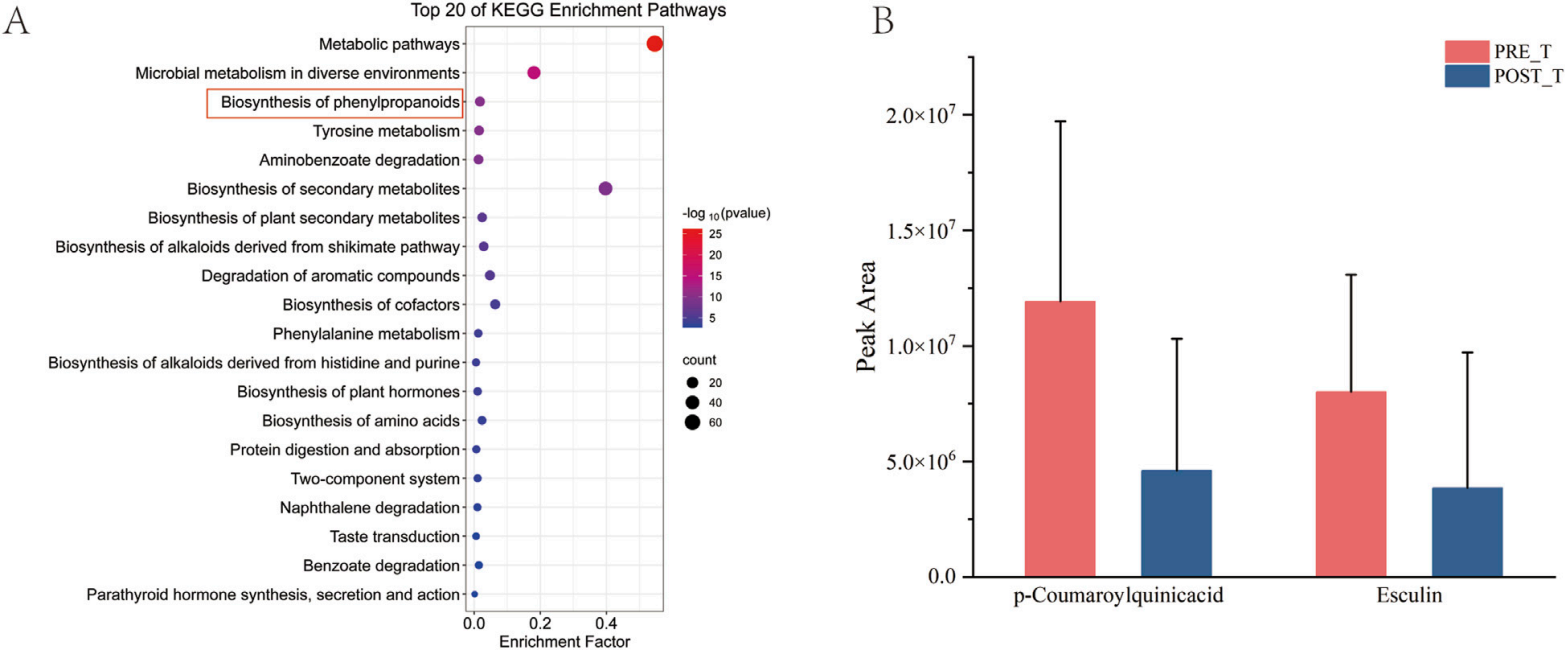
Analysis of differential pathways. **(A)** Bubble map of KEGG enrichment analysis for differential metabolites. **(B)** Histogram of quantitative outcomes of p-Coumaroylquinic acid and Esculin before and after treatment.

After the KEGG enrichment analysis of differential metabolites, the first 20 enrichment pathways were presented (Figure 9A). It mainly involves the biosynthesis of phenylpropanoids, Tyrosine metabolism, and the biosynthesis of amino acids. The biosynthesis of phenylpropanoids is one of the key differential metabolic pathways. Metabolites related to the biosynthesis of phenylpropanoids, such as p-Coumaroylquinic acid and Esculin, decreased significantly after treatment (*P* < 0.05) (Figure 9B).

### 3.4 Correlation analysis of sore flora and metabolites

**FIGURE 10:**
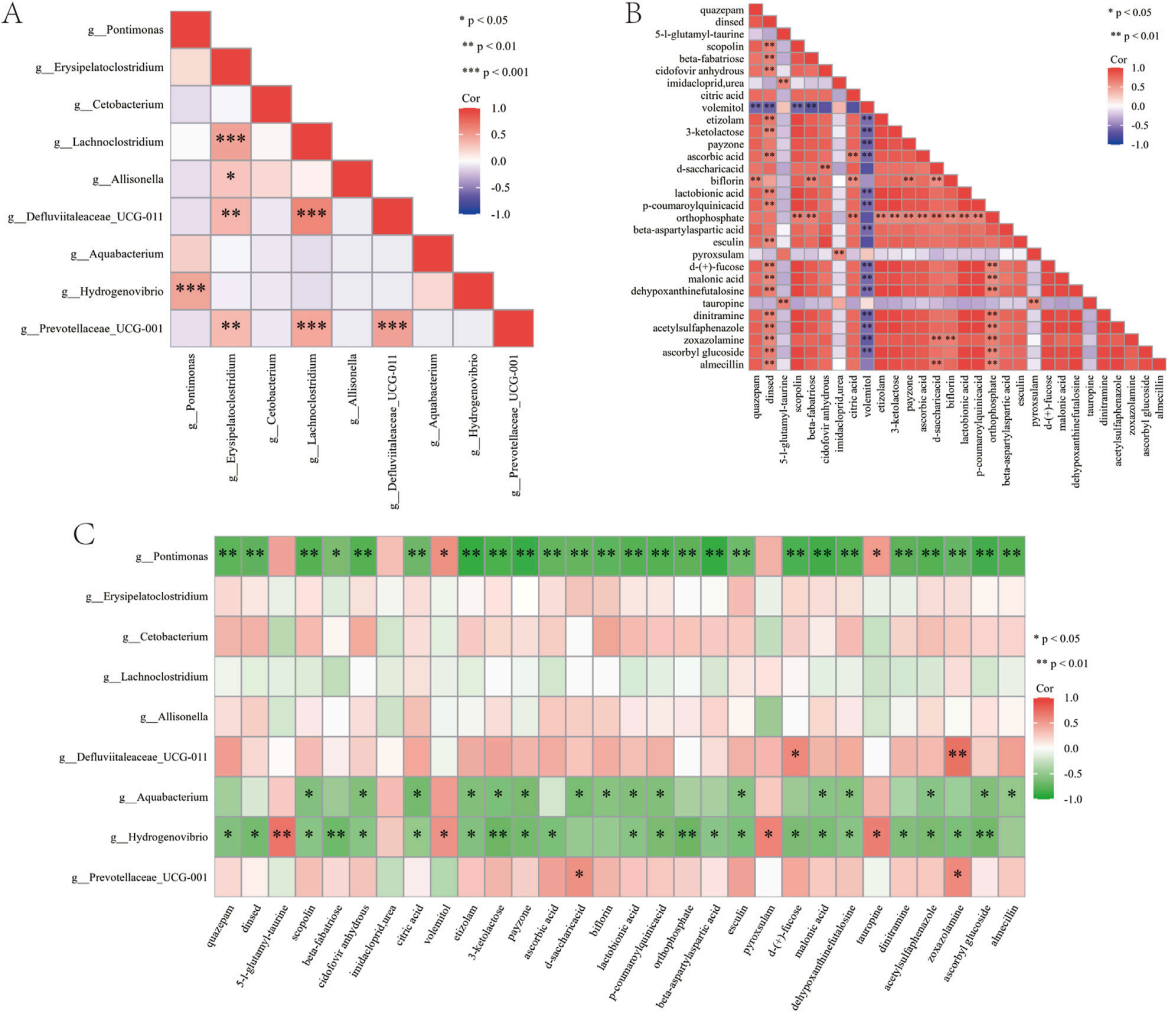
Correlation analysis of sore flora and metabolites. **(A)** Correlation analysis of differential genus-level microbial taxa. **(B)** Correlation analysis of differential metabolites. **(C)** Correlation analysis between differential microbial taxa and differential metabolites. “*” indicates *P* < 0.05.

In this study, correlation analysis takes *P* < 0.05 and ∣r∣> 0.6 as significance criteria. The analysis of differential genus-level microbial taxa before and after treatment with the Huafu Zaisheng method revealed that Defluviitaleaceae_UCG-011 and Lachnoclostridium exhibited the strongest positive correlation (Figure 10A). Further analysis of differential metabolites before and after treatment showed that scopolin and lactobionic acid exhibited the strongest positive correlation (Figure 10B). In the correlation analysis between differential genera and metabolites, Pontimonas, Hydrogenovibrio, and Aquabacterium showed a significant negative correlation with most differential metabolites, suggesting a pivotal role in metabolite degradation or the regulation of metabolic balance (Figure 10C).

## 4 Discussion

This study, based on clinical research, investigates the efficacy of Huafu Zaisheng method in treating diabetic foot tendon-exposed wounds and explores the mechanisms of microbial communities and metabolites through 16S rDNA sequencing and untargeted metabolomics. The findings indicated that the treatment markedly enhanced the formation of granulation tissue and expedited the healing process, thereby substantiating its clinical efficacy to a considerable extent. Bromelain, a complex mixture of proteolytic enzymes including various proteases such as stem bromelain, fruit bromelain, and ananain, has been demonstrated to promote wound healing through multiple mechanisms, including anti-inflammatory, antioxidant, fibrinolytic, and immunomodulatory effects (Hale et al., 2005; Azarkan et al., 2020). Based on preliminary UPLC fingerprinting analysis, Shengji Ointment contains bioactive components such as azelaic acid, 3-N-butyl phthalate, n-butylphthalide, palmitoleic acid, linolenic acid, ligustilide, linoleic acid, palmitic acid, and oleic acid, which may synergistically contribute to its wound-healing properties (Zhao et al., 2023b). The synergistic wound-healing mechanism of bromelain and Shengji ointment may involve multifaceted cooperative effects: On one hand, the proteolytic activity of bromelain effectively degrades necrotic tissue in wounds, reducing its physical barrier effect, thereby significantly enhancing the penetration of active components in Shengji ointment. On the other hand, Shengji ointment stimulates nutrient secretion and creates a moist wound environment, which not only helps maintain bromelain’s bioactivity but also promotes liquefaction and drainage of necrotic tissue. Notably, existing research has demonstrated that nitric oxide (NO) plays pivotal regulatory roles in diabetic wound healing, including vascular homeostasis modulation, anti-inflammatory effects, and antibacterial activity (Malone-Povolny et al., 2019). Our preliminary experimental results indicate that the combination of bromelain and Shengji ointment significantly increases NO production at wound sites compared to mono-therapy groups, suggesting this phenomenon may serve as a key molecular mechanism underlying their synergistic wound-healing effects (Zhang et al., 2012a). The treatment altered the composition of the local microbial community in treating diabetic foot ulcers with tendon exposure, resulting in a significant reduction of potential pathogenic bacteria and an increase in potentially beneficial bacteria. Anaerobic bacteria, commonly present in deeper lesions, are often linked to fever, foul-smelling wounds, and ulcer depth and duration (Sasikumar et al., 2018; Villa et al., 2024). The observed reduction in anaerobic bacteria, such as Thiotrichales and Defluviitaleaceae, following treatment may suggest a decrease in bacterial virulence and potentially a reduction in wound depth (Coluccio et al., 2024). Erysipelatoclostridium is closely associated with tissue inflammation and severe infections caused by compromised immunity (Zakham et al., 2019; Milosavljevic et al., 2021). Its reduction in wound tissue after treatment may help alleviate local inflammatory responses, thereby enhancing the environment for tissue repair. Previous research has indicated that Lachnoclostridium is positively correlated with the risk of type 2 diabetes. (Li and Li, 2023). Cetobacterium exhibits strong carbohydrate metabolism capabilities (Garibay-Valdez et al., 2024). The reduction in Lachnoclostridium and the increase in Cetobacterium may indicate a shift in the microbial community from a pathological to a healthier state. However, the specific functions of certain bacterial groups, including Defluviitaleaceae, Piscirickettsiaceae, Pontimonas, Aquabacterium, Hydrogenovibrio, and Prevotellaceae_UCG-001, remain insufficiently evidenced. The PICRUSt2 functional prediction analysis indicated that the Huafu Zaisheng method significantly altered the functional potential of wound microbiota. Notably, pathways like the tricarboxylic acid cycle VII and pyruvate fermentation to butanoate were upregulated, whereas mannose biosynthesis was downregulated. These changes suggest that these pathways may be critical to how the Huafu Zaisheng method promotes healing of diabetic tendon-exposed wounds, potentially offering valuable targets for future mechanistic investigations.

Non-targeted metabolomics were used to evaluate the changes of wound metabolites before and after the treatment of decomposition and regeneration. We focus on the first 30 different metabolites. KEGG enrichment analysis shows that different metabolites mainly involve the biosynthesis of phenylpropanoid, Tyrosine metabolism, biosynthesis of amino acids and other pathways. A study has demonstrated that phenylpyruvate, an essential intermediate metabolite in the phenylpropanoid biosynthesis pathway, is significantly accumulated in diabetic foot ulcers. This accumulation contributes to the perpetuation of chronic inflammation and impaired diabetic wound healing by enhancing inflammasome activity and the release of proinflammatory factors (Lv et al., 2023). Based on enrichment results and literature reports, the Huafu Zaisheng method may regulate phenylpyruvate metabolism to alleviate chronic inflammation, while also influencing the overall pathway of phenylpropanoid biosynthesis, thereby optimizing the metabolic microenvironment of tendon-exposed wounds in diabetic foot ulcers and promoting wound healing and tissue regeneration. Amino acids serve as the fundamental building blocks for protein synthesis. They contribute to the production of structural proteins like collagen, which supports the formation of new tissues (Barbul, 2008). Additionally, amino acids play a critical role in regulating cellular metabolic pathways, thereby facilitating the proliferation and migration of fibroblasts and keratinocytes. These processes collectively promote wound healing and tissue regeneration (Kim et al., 2015; Albaugh et al., 2017). The enrichment results of this study suggest that enhancing amino acid biosynthesis may represent a key mechanism by which the Huafu Zaisheng method promotes wound healing. A study has indicated that key genes of tyrosine metabolism, namely, tyrosinase (TYR), tyrosinase-related protein 1 (TYRP1) and dopachrome tautomerase (DCT), are involved in melanogenesis and play an important role in the acute phase of wound healing (Zhu et al., 2021). This study also revealed notable alterations in the tyrosine metabolic pathway, indicating that the Huafu Zaisheng method may potentially target this pathway to facilitate the healing of diabetic foot tendon-exposed wounds.

The study identified a significant reduction in the abundance of Pontimonas, Hydrogenovibrio, and Aquabacterium on the wound following treatment, along with a notable negative correlation between these bacteria and the majority of the differential metabolites. The results of this study indicate that the Huafu Zaisheng method may facilitate wound healing and tissue regeneration by influencing the structure of the microbial community and mitigating the detrimental effects of specific bacteria on metabolites, thereby enhancing the metabolic microenvironment. Further investigation is required to elucidate the precise function of these flora in the metabolic regulation of tendon-exposed wounds in diabetic feet, as well as to identify potential therapeutic targets.

The limitations of this study should be acknowledged. First, the lack of follow-up data precludes evaluation of the intervention’s long-term efficacy and safety, necessitating further investigation. Moreover, while wound exudate analysis provides insights, it may not fully reflect the wound tissue’s metabolic profile or complete microbial composition. An important limitation is the absence of microbial/metabolomic data from hydrogel-treated controls. Previous research has confirmed that certain bioactive hydrogels can increase beneficial microbiota (e.g., Corynebacterium_1 and *Lactobacillus*) and restore skin microbial balance in diabetic wounds (Hao et al., 2022). Consequently, our inability to compare microbial profiles between treatment groups may obscure potential differences in wound microenvironment modulation and hinder definitive conclusions about our intervention’s specific effects. Additionally, the study population was restricted to a single center with specific inclusion criteria, which may limit the generalisability of findings to broader clinical settings or diverse patient groups. Another limitation is that some differential microbiota and metabolites lack robust literature support, hindering interpretation of their biological relevance. Critically, correlation analyses cannot establish causality between microbial/metabolite changes and therapeutic effects. Future studies should employ targeted metabolomics and animal models to address these mechanistic gaps.

## 5 Conclusion

Generally, the treatment of tendon-exposed wounds of diabetic foot by Huafu Zaisheng method may facilitate wound healing by regulating the composition of the bacterial community and the metabolic environment. This study concentrated on the alterations in microflora at the generic level. The potential pathogenic bacteria (Erysipelatoclostridium, Lachnoclostridium, Defluviitaleaceae_UCG-011, Pontimonas, Aquabacterium, Hydrogenovibrio, Prevotellaceae_UCG-001) exhibited a notable decline, whereas the possible beneficial bacteria (Cetobacterium and Allisonella) demonstrated a marked increase. The regulation of pivotal metabolic pathways, including phenylpropanoid biosynthesis, tyrosine metabolism, and amino acid biosynthesis, may serve as a mechanism for facilitating healing. In particular, the reduction in the abundance of Pontimonas, Hydrogenovibrio, and Aquabacterium upregulates the expression of beneficial metabolites and thus promotes wound healing. These findings offer new insights into the mechanism of treatment for tendon-exposed wounds of the diabetic foot by Huafu Zaisheng method.

## Data Availability

The metabolomics data presented in this study have been deposited in the Metabolights repository under accession number MTBLS12713, and the 16S r RNA gene sequencing data have been deposited in the NCBI Sequence Read Archive (SRA) under accession number PRJNA1290628.
