# Supplementary material for: A randomized controlled trial of Shengji ointment combined with bromelain in promoting healing of tendon-exposed diabetic foot wounds: integrated 16S rDNA sequencing and metabolomics analysis
Source: Front Pharmacol. 2025 Nov 7;16:1666278. doi: 10.3389/fphar.2025.1666278 (PMC12634329; doi:10.3389/fphar.2025.1666278)
Supplement: Supplementary file 1 [file Supplementaryfile1.docx]

| 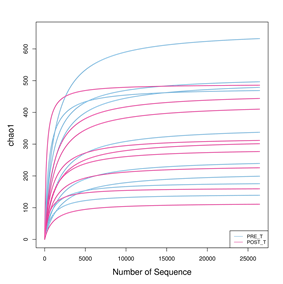 | 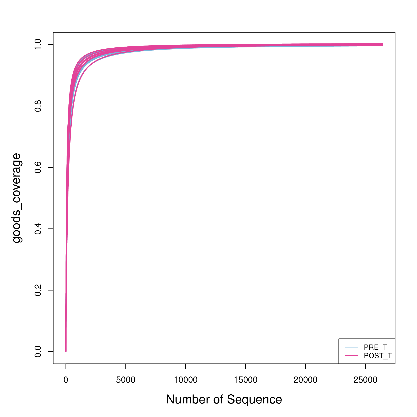 | 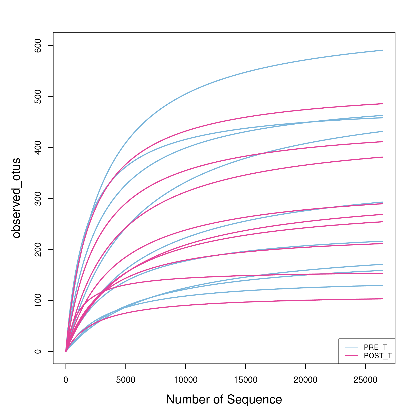 |
| --- | --- | --- |
| Chao1 | Goods_coverage | Observed species |
| 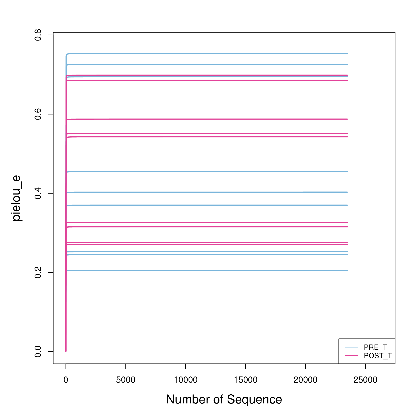 | 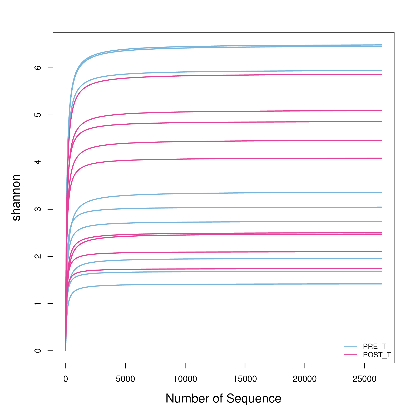 | 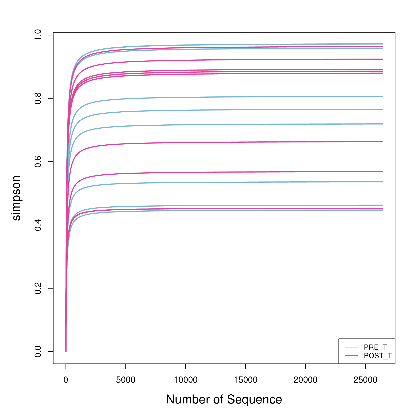 |
| Pielou-e | Shannon | Simpson |
| Rarefaction curves. The x-axis represents the number of randomly selected sequences, and the y-axis represents the exponent value for each sample when the same number of sequences is extracted. | | |
